# Genetic improvement and bioactive potential of rice bean (*Vigna umbellata*) for climate resilience and sustainable agriculture

**DOI:** 10.3389/fpls.2026.1817491

**Published:** 2026-04-30

**Authors:** Raymond Malinda Lutege, Philip C. Stevenson, Caspar C. C. Chater, Pavithravani Venkataramana

**Affiliations:** 1 School of Life Sciences and Bioengineering (LiSBE), The Nelson Mandela African Institution of Science and Technology (NM-AIST), Arusha, Tanzania; 2 Traits Diversity and Functions Royal Botanic Gardens, Kew, Richmond, United Kingdom; 3 Natural Resources Institute, University of Greenwich, Chatham, United Kingdom; 4 Plants, Photosynthesis and Soil, School of Biosciences, The University of Sheffield, Sheffield, United Kingdom

**Keywords:** *Vigna umbellata*, antioxidant rice bean, biotic and abiotic tolerance, breeding approaches, germplasm conservation, underutilised crop

## Abstract

The integration of underutilised orphan crops into food systems can reduce the reliance on major crops for global food security and support adaptation to climate change. Major crops are increasingly susceptible to climate effects such as drought, warming temperatures, floods, and unfavourable growing conditions. Rice bean, for example, is a minor crop that is well adapted to hot, dry climates and is highly nutritious, high-yielding, and resistant to various biotic stresses. Rice bean also contains bioactive compounds like phenolic acids and flavonoids that confer resistance to insects and diseases but they also have health benefits. Despite these advantages, rice bean faces adoption barriers linked to traits such as pod shattering, indeterminate growth, lengthy cooking times due to seed hardness, and untimely pod maturity. Therefore, utilising modern tools to explore its genomic and bioactive potential is essential, especially given the limited literature on rice bean use across different regions. This review examines current research on rice bean’s genetic diversity, nutritional value, environmental tolerance, bioactive properties, and breeding strategies, aiming to unlock the crop’s full potential. This will guide breeding efforts, inform future studies, and provide solutions for leveraging rice bean’s inherent qualities and transferring knowledge to other crops. Integrating agro-morphological traits, bioactive potentials, and genomic resources through advanced breeding techniques could significantly boost rice bean’s global adoption across diverse regions, enabling region-specific climate adaptation in breeding programs.

## 1 Introduction

Rice bean (*Vigna umbellata* (Thunb.) Ohwi and Ohashi) is an underutilised annual, self-pollinated warm seasonal legume, native to and widely cultivated in South and Southeast Asia. It is diploid, with 2n = 22 chromosomes (Seehalak et al., 2006) and a draft genome size of 414 Mb (Kaul et al., 2022). Rice beans grow to 3 meters, with a branching stem, and they are characterised by an erect, semi-erect, or twining growth habit. They have trifoliate leaves measuring 6–9 cm long, with bright yellow flowers that appear in clusters (Roomesh Kumar Jena et al., 2024). The crop is a primary protein source for human and livestock (Sanchit et al., 2023). It is also used in cropping systems as green manure and a cover crop to enhance soil fertility and promote sustainable agricultural practices (Katoch, 2011; Sanchit et al., 2023). Its dry grains are rich in protein (18.3–25%) with high *in vitro* digestibility (59–93%). It has a well-rounded amino acid profile (0.90–17 mg/100 g), minerals (mg/100 g) of 325, 1463, 491.58, 326.87 and 488.78 for Na, K, Ca, Mg and P, respectively, ash (4.26%), fibre (4.8%), and minimal fat (< 1%) (Kaur et al., 2013; Pattanayak et al., 2019).

Rice beans are well adapted to high-temperature environments, exhibit resistance to bruchid attack, limited water stress, and thrive well on poor soils with a broad range of soil pH levels, thereby making them a suitable choice for resource constrained small scale farmers (Kumar et al., 2014). The world production index for rice beans is not included in the FAO databases but is combined with other beans in the “beans, dry” category due to its limited use (Sherasia et al., 2017). However, rice bean has high production potential in India. For example, specifically in the Nagaland region during the year 2020/21, grain production was recorded at 1.1 t/ha (Kuotsu et al., 2023), higher than other *Vigna* species, such as cowpea, which has a global production of 0.5 t/ha (Sindhu et al., 2019). The crop encounters adoption challenges due to its intrinsic characteristics, such as photoperiod sensitivity, indeterminate growth, sporadic and asynchronous flowering, dehiscent pods, small and hard seeds, and pod shattering (Pattanayak et al., 2019; Parker et al., 2021). Conventional breeding has been used to improve rice bean in terms of agronomic and nutritional performance; however, the results revealed moderate levels of polymorphism (Shafiqul et al., 2017) a limited number of domestication loci (Isemura et al., 2010), low fertility and unstable progeny (Bhanu, 2018), and the transfer of undesirable traits (Andersen, 2012).

To develop rice bean varieties resistant to low water stress, with reduced pod shattering, and favoured by both farmers and consumers, breeding techniques such as marker-assisted breeding, genomic selection, and transgenic methods can be used. Recent reviews emphasise the use of molecular markers, genetic diversity studies, map construction, QTL identification, and the implementation of marker-assisted breeding (Guan et al., 2022; Francis et al., 2023). This review centres on bioactive potential, genetic improvement strategies aimed at climate resilience and sustainability and focuses on key adaptation traits, breeding methods, and future plans to develop rice bean varieties ready for climate change in farming systems.

## 2 Genetic diversity for climate-resilient rice bean breeding

Genetic diversity in rice bean is centered on wild, landrace and cultivated varieties, with wild varieties noted to have the highest level of variation within a population. Molecular studies on SSR and SNP markers revealed that cultivated varieties contribute a small fraction of the genetic variation in the rice bean population compared to landraces and wild varieties, which harbour potential diversity traits for tolerance to limited water stress, early maturity, and moderate resistance to pests and diseases.

*Vigna* species germplasm collections exist worldwide, including cultivated and wild varieties. The largest genebanks collection is the International Institute Tropical Agriculture (IITA) in Nigeria (NGA039), followed by the World Vegetable centre in Taiwan (TWN001), ICAR’s -National Bureau of Plant Genetic Resources in India (IND001), the United States Department of Agriculture (USDA) in the USA (USA016), and National Agriculture and Food Research Organization in Japan (JPN183) (Nair et al., 2023). Genebank collections play a key role as sources of breeding materials for plant architecture, seed features, pests, and pathogens.

A genome-wide association study (GWAS) involving 440 rice bean accessions identified three geographical patterns: South and Southeast Asia, South China, and North China. The landrace varieties demonstrated adaptability to both low-latitude (18-22°N) and high-latitude (40°N) environments. These findings can be used to guide breeding programs targeting specific agro-ecological zones that could benefit from regionally adapted alleles, as indicated by this spatial structuring (Guan et al., 2022). Additionally, 13 SSR markers were utilised to assess 472 rice bean samples from 16 Asian countries grown in a greenhouse, including both cultivated and wild types. Results confirmed that wild varieties exhibit slightly higher gene diversity (0.678) than cultivated varieties (0.565). This shows that the wild varieties constitute a substantial reservoir of new alleles that are presently underutilised in breeding programs (Tian et al., 2013). The same study used 44 SSR markers to evaluate 40 rice bean samples grown in the field and found limited genetic polymorphism in seven of the 44 markers. However, the dendrogram showed two clusters, with Cluster I containing 37 members and Cluster II containing 3, whilst a structure analysis divided the population into four groups with 5, 23, 7 and 5 genotypes. This genetic structure indicates the importance of selecting parents from genetically distant groups to enhance heterosis and broaden the genetic base of improved varieties (Thakur et al., 2017). In addition, three clusters were identified from the analysis of 120 rice bean genotypes. Cluster I showed the highest mean yield (Devi et al., 2022). This shows that cluster-based selection can be an effective method for finding genotypes with high yields whilst preserving genetic diversity. Genetic studies on rice bean domestication show that important agronomic traits are influenced by several quantitative trait loci (**Table 1**) spread across multiple linkage groups (LG2, LG4, and LG7), with a significant locus on LG4. This region relates to various domestication traits, such as plant structure, flowering patterns, and seed and pod features (Isemura et al., 2010). The overlap of these traits indicates that domestication is controlled by clustered QTL regions, possibly involving pleiotropy and coordinated genetic regulation (Guan et al., 2022; Tyagi et al., 2024). This arrangement suggests that natural or artificial selection during domestication likely targeted multiple traits simultaneously, offering opportunities to efficiently improve rice bean through marker-assisted selection and to develop cultivars that are resilient to climate change. These genetic diversity studies collectively indicated the valuable potential of wild relatives and landraces as sources of novel adaptive traits to improve disease resistance, yield stability, and drought tolerance. The higher genetic variability found in wild populations than in cultivated ones indicates that domestication has reduced the genetic diversity of cultivated cultivars, possibly limiting their ability to adapt to changing climate conditions, which imposes the need to utilise wild relatives for breeding of stable cultivars (Tian et al., 2013). Despite the availability of genetic diversity data and molecular markers, their practical application in rice bean breeding remains limited. Future research should focus on integrating genomic resources with phenotypic assessments across diverse germplasm collections to accelerate the development of climate-resilient rice bean varieties.

**Table 1:** Summary of identified QTLs linked to domestication and resistance traits to pests and diseases in rice beans.

Trait category	QTL/locus description	Genomic location	Associated function	Reference(s)
Pod dehiscence	*Pdt4.7.1*	LG7	Pods twists	(Isemura et al., 2010)
Seed size	Seed-related QTLs (domestication loci)	LG4 (major), LG2	Seed size, seed coat, dormancy	(Isemura et al., 2010)
Pod traits	Pod-related QTLs (*Pdw4.4.1, Pdl4.4.1*)	LG4	Pod length and width	(Isemura et al., 2010)
Leaf size	Leaf related traits (*Lfml4.4.1, Lfmw4.4.1, LFPL* and *LFPW* not detected)	LG4	Maximum leaf length, width and primary leaf length and width	(Isemura et al., 2010)
Plant architecture	*Brn4.4.1*	LG4	Stem growth, plant form	(Isemura et al., 2010)
Flowering traits, adaptation, and yield	*FUL (FRUITFULL), FT (FLOWERING LOCUS T)*, and *PRR3 (PSEUDO-RESPONSE REGULATOR 3)*	Multiple genomic regions	Photoperiod response, flowering time and Seed size	(Guan et al., 2022)
Bruchid resistance	11 QTL's Resistance (*Cmtdp1.1, Cmpd1.1, Cmpd1.5,Cmpd1.4,Cmpd1.6, Cmtdp1.2,Cmpd1.7,Cmae1.1,Cmpd1.2, Cmpd1.3, Cmpd1.2*)	Multiple linkage groups	Resistance to storage pests	(Venkataramana et al., 2016)
MYMV resistance	*qMYMV4_1, qMYMV5_1, qMYMV6_1, qMYMV10_1, qMYMIV6.1.1*	Major genomic regions identified	Virus resistance	(Mathivathana et al., 2019; Dhaliwal et al., 2022)

## 3 Source of nutrition and production traits

Adopting rice beans could decrease reliance on major pulses such as common beans, cowpea, and chickpeas. It has been reported that proteins, minerals and essential amino acids vary amongst *Vigna* species and other major pulses as detailed in **Table 2**. Several studies have reported variations in rice bean nutritional composition, including protein (18% – 25%), carbohydrate (44% – 65%), fibre (1.9% – 4.2%), fat (3.46 – 4.03%), and ash (3.99% – 4.58%) (Rodriguez and Mendoza, 1991; Katoch, 2013; Katoch et al., 2023). This variability makes rice beans a competitive source of vitamins and protein compared with other major pulses, with the added benefit of reported high genetic diversity that can be leveraged to improve nutrition.

**Table 2:** Nutritional composition, mineral content, and the major phenolic profile of rice bean (*Vigna umbellata*).

Crop	Common name	Scientific name	Protein (%)	Carbohydrates (%)	Fibre (%)	Fat (%)	Ca (mg/ 100 g)	Fe (mg/ 100 g)	P (mg/ 100 g)	Lysine (g/100 g)	Leucine (g/100 g)		Threonine (g/100 g)	Isoleucine (g/100 g)	References
Major legumes	Pigeon pea	*Cajanus cajan*	21.70	62.78	1.50	0.38	11.60	4.50	348.60	1.52		1.55	0.77	0.79	(Saxena et al., 2010; Kuraz Abebe, 2022)
	Cowpea	*Vigna unguiculata*	23.40	55.00	3.90	8.30	89.00	10.50	468.00	5.50		7.00	4.00	4.00	(Jayathilake et al., 2018; Enyiukwu et al., 2020)
	Chickpea	*Cicer arietinum*	25.00	50.10	3.90	–	170.00	–	390.00	6.60		7.10	3.40	3.80	(Begum et al., 2023)
	Common beans	*Phaseolus vulgaris*	25.00	65.25	7.33	2.35	438.00	28.22	1207.00	2.20		0.49	0.34	0.32	(Chávez-Servia et al., 2016; Tc, 2018)
Underutilised legumes	Rice bean	*Vigna umbellata*	25.00	60.00	4.80	1.40	354.50	6.45	34.60	1.64		1.22	7.30	1.03	(Pattanayak et al., 2019; Dwivedi et al., 2023)
	Mung bean	*Vigna radiata*	23.60	–	3.30	1.00	199.00	7.50	313.00	0.06		0.07	0.03	0.03	(Wang, 2025)
	Urd bean	*Vigna mungo*	23.40	–	3.80	1.00	–	–	–	0.00		0.00	0.00	0.00	(Gore et al., 2025)
	Adzuki bean	*Vigna angularis*	22.80	44.60	12.70	0.80	66.00	5.00	381.00	0.85		0.87	0.37	0.50	(Wang et al., 2022)
	Bambara groundnut	*Vigna subterranea*	21.00	61.00	8.50	7.50	79.00	5.50	322.00	0.11		1.39	0.62	0.78	(Maphosa et al., 2022)
	Moth bean	*Vigna aconitifolia*	24.00	63.80	–	1.60	647.00	13.00	360.00	1.25		1.54	0.00	1.14	(Bhadkaria et al., 2022)

In relation to other *Vigna* species, a GWAS study in Cowpea identified single-nucleotide polymorphisms (SNPs) and a candidate gene (*Vigun08g039200*) associated with seed protein content (Chen et al., 2023). This implies that genomic regions influence protein levels in a *Vigna* species. Also, high heritabilities of 97.77% for yield and 93.41% for protein content were confirmed in Cowpea (Nautiyal et al., 2024). Production efficiency, as measured by yield, was improved through backcrossing, using rice bean (RBL1) as the male parent with a good production index and black gram as the female parent. The results show an increase in yield from -35.48% to 50.31%. It was observed that rice bean genes were successfully integrated into black gram, leading to traits such as an increase in pods per plant from 32 to 42 and in 100-seed weight from 4.5 g to 5 g (Singh et al., 2013). These changes in yield-related traits directly influence the overall nutritional output per unit area, since increased seed quantity and weight lead to better availability of protein and minerals. Additionally, the genetic similarity between rice bean and other *Vigna* species relative to other pulses, such as common beans, offers opportunities to transfer knowledge from one species to hasten genetic enhancement in the other (Pattanayak et al., 2019). Furthermore, the successful introgression of genes from rice beans into other *Vigna* species shows rice beans’ potential as a donor crop for genetic improvement. Identifying genes regulating essential traits in rice beans, such as flowering time and yield, could aid breeding for climate-adapted varieties (Boukar et al., 2020; Guan et al., 2022). Moreover, the genotype × environment interaction showed that rice beans can perform well across different latitudes, which could help breed varieties suited to specific regions.

The reported differences in the nutritional profile of rice bean accessions have ramifications for breeding and agricultural improvement. Rice bean has significant genetic potential to enhance the nutritional quality of grain legumes due to its relatively high protein content (18–25%), balanced amino acid composition, and mineral richness (Katoch, 2013). The observed variations between accessions imply that these characteristics are genetically controlled and may be successfully used in genomic-assisted breeding and selection. Specifically, protein concentration, mineral accumulation, and key amino acids such as leucine and lysine are suitable targets for biofortification programs aimed at improving nutritional quality (Ofori et al., 2022). Additionally, rice bean germplasm collections may be useful sources of alleles to enhance nutritional qualities, both within rice bean breeding programs and across related *Vigna* species, given the significant genetic heterogeneity observed for these traits. Therefore, the development of rice bean cultivars that combine high yield, improved nutritional value, and tolerance to climate-related stresses could be accelerated by integrating nutritional trait evaluation with genetic methods such as GWAS and marker-assisted selection. However, in terms of diet, rice beans provide significant protein to help individuals meet their daily protein requirements, especially in low-income regions where plant-based protein sources are vital. Its mineral content, including calcium and iron, also supports nutritional security, particularly for populations vulnerable to micronutrient deficiencies. These findings show that whilst rice beans hold great nutritional potential, there remains a notable gap in developing these qualities into well-characterised and widely adopted cultivars.

## 4 Breeding opportunities for climate resilience

### 4.1 Biotic stress tolerance

Diseases and insect pests constrain crop production at both the vegetative and reproductive stages of plants (Dresselhaus and Hückelhoven, 2018). For example, bruchid weevils are key storage pests, whilst field pests such as thrips, whitefly, jassids, and pod borers cause significant losses in many leguminous crops, particularly those in the genus *Vigna.* These pests lay eggs and, upon hatching, produce larvae that feed on leaves or penetrate seeds or pods to complete their development, causing extensive damage. This leads to seed weight loss, lower germination rates, and substantial post-harvest losses. Major bruchid pests threatening crops are *Callosobruchus maculatus*, *Callosobruchus chinensis*, and *Callosobruchus analis* (Ragul and Manivannan, 2021).

Insect resistance has been studied in rice bean (**Table 3**) and is considered an important trait for improved storage and postharvest stability (Aidbhavi et al., 2021). Studies have shown that antibiosis mechanisms mediated by antinutritional compounds, such as trypsin inhibitors and the cysteine protease enzymes, inhibit larval growth of *Callosobruchus* spp. within the cotyledons (Kasettranan et al., 2010; Kaur and Gill, 2016). This is supported by a study showing increased trypsin and protease inhibitor activity (1576–3120 TIU g^-^¹ and 2061–4923 CPIU g^-^¹), respectively, in rice bean accessions, which has been linked to reduced bruchid development (Srinivasan and Durairaj, 2007). Additionally, bruchid resistance is quantitatively inherited at the molecular level and is controlled by multiple quantitative trait loci (QTLs) distributed across multiple linkage groups (Mishra et al., 2018). The QTL mapping study has identified 11 QTL loci across 10 linkage groups in multiple genomic regions associated with resistance to *C. maculatus* and *C. chinensis*, suggesting polygenic regulation of the trait (Venkataramana et al., 2016). The distribution of these QTLs throughout the genome indicates that resistance is stable yet complex, offering opportunities for genetic improvement (**Table 1**). Also, the possibility of resistance genes introgressing into susceptible cultivars is demonstrated by the successful transfer of bruchid resistance from rice beans to allied *Vigna* species such as Azuki bean (Katna et al., 2024). The presence of QTL linked to bruchid resistance paves the way for utilising marker-assisted selection (MAS) to breed rice bean with enhanced agronomic traits. Collectively, all of these studies emphasise the importance of combining breeding, genetic, and biochemical methods to improve grain legumes’ long-term resistance to storage pests.

**Table 3:** Rice bean genotypes known to be resistant to pests.

Pest name	Category	Genotype	Mode of evaluation	References
*Callosobruchus analis*	Storage pest	IC251439, IC251442, PRR 2007-2, IC251440, TCR 279, JP99485, JP100304, JP100311	Bioassay	(Kashiwaba et al., 2003; Singh et al., 2022)
*Callosobruchus maculatus*	Storage pest	LRB238, JP100304, RBL-6, JP99485, JP100311, LRB161, LRB1, KHRB1, LRB295, LRB168, IC-524075, IC-341983	Bioassay	(Pavithravani et al., 2013; Venkataramana et al., 2016; Vishwas and Deshpande, 2018)
*Callosobruchus chinensis*	Storage pest	JP99485, JP100304, JP100311	Bioassay	(Kashiwaba et al., 2003)
Thrips, whitefly, jassid, and pod borer	Field pest	BRB 15-2, BRB 4, BRB 31, BRB 47, LRB 589, LRB 584, RBL-35	Field trial	(Cheema et al., 2019)

Rice beans exhibit resistance to diseases such as Yellow Vein Mosaic Virus (YVMV) (**Table 4**), which poses a significant threat to leguminous crops (Guan et al., 2022). Whiteflies (*Bemisia tabaci*) are the primary vectors of YVMV, which is caused by begomoviruses that infect plants and disrupt photosynthesis and chloroplast functions, resulting in distinctive yellowing symptoms (Nair et al., 2023; P et al., 2024). Various methodologies have effectively integrated resistance genes from rice beans into other susceptible *Vigna* species. Rice beans are cross-compatible with *Vigna mungo*, and *V. radiata* var. *Sublobata.* For example, rice beans and mung bean *(V. radiata*) have been crossed successfully to develop varieties resistant to Mung bean Yellow Mosaic Virus (MYMV) (Sudha et al., 2013). This was later confirmed by a study in which the interspecific population between rice bean and mung bean revealed four QTLs (designated as *qMYMV4_1, qMYMV5_1, qMYMV6_1*, and *qMYMV10_1*) conferring resistance to MYMV and the QTL on chromosomal 4 (*qMYMV4_1*) was highly conserved in all populations (Mathivathana et al., 2019). The resistance to MYMV in rice bean is associated with genetic traits (loci) that restrict the virus’s capacity to replicate and disperse within host tissues (Jha et al., 2023). A similar study identified resistance to MYMV in black gram by using rice bean as a source to introduce the QTL (*qMYMIV6.1.1*) that provides resistance to MYMV (Dhaliwal et al., 2022).

**Table 4:** Rice bean genotypes resistant to diseases.

Diseases	Disease category	Genotype	Evidence	References
MYMV	Virus	RBL1, RBL6, RBL 9, RBL35, RBL50, TNAU RED, Resplant3, Megha Rumbaija 1 (RCRB 1-6)	Donor parent, Field screening	(Sehrawat et al., 2016; Bhanu et al., 2017; Mathivathana et al., 2019; Mishra et al., 2020; Pandiyan et al., 2020)
*Cercospora* leaf spot	fungi	RBL1, PRR-1, PRR-2 and VRB-3	Resistance observed in hybrids	(Singh et al., 2013; Sudha et al., 2013)
Powdery mildew	fungi	R200	Field screening	(Kitomari et al., 2024)
Bacterial leaf blight	Bacterial	RBL1	Field screening	(Singh et al., 2013; Bhanu et al., 2017)

The above studies demonstrate that interspecific hybrids of urd and rice bean exhibit resistance to *Cercospora* leaf spot, a trait conferred by rice bean as the donor parent (Sharma et al., 2022). Furthermore, rice bean demonstrated resistance to powdery mildew during field evaluations conducted on mung bean, where rice bean was used as a control (Singh et al., 2013). However, there has been limited research on the durability of these resistance traits across various genetic backgrounds and environments. Additionally, a comparable study identified two QTLs in mung bean responsible for powdery mildew resistance, which were sourced from rice bean (Singh et al., 2022). Studies have shown that rice bean is an important source and donor of resistance genes for improving tolerance to pests and diseases in *Vigna* crops (Mishra et al., 2018, Mishra et al., 2020). This implies that multiple loci, including major- and minor-effect QTLs, are likely responsible for rice bean resistance, and that these loci act together to confer resistance. The successful transfer of resistance loci to related species like mung bean and black gram demonstrates the potential of rice bean germplasm to expand the genetic diversity of cultivated legumes (Sehrawat et al., 2016). However, despite the identification of several resistance loci, they remain without direct functional validation and characterisation in rice beans. Hence, the development of climate-resilient *Vigna* cultivars with enhanced resistance to new pest and disease challenges may be facilitated through molecular breeding and interspecific hybridisation. This suggests that rice bean’s ability to resist pests and diseases is primarily attributable to its diverse genetic composition and the presence of transferable alleles at resistance loci.

### 4.2 Abiotic stress tolerance

#### 4.2.1 Drought stress response

Rice bean exhibits appreciable drought-tolerance and is consequently mostly cultivated on marginal lands in rain-fed cropping systems, requiring minimal fertilizer and weed control (Khatun et al., 2021). Apart from the evidence that rice beans have the potential to adapt to drought conditions, the existing literature on specific drought QTLs for rice bean, the diversity of physiological traits across landraces, and breeding efforts for drought tolerance remain limited.

A physiological and biochemical study revealed that the germination percentage of rice bean was generally high (up to 100%) under moderate drought conditions with 10% polyethylene glycol (PEG 6000), salinity at 200 mM NaCl, and heavy metal treatments (Cu 50-200 µM and Pb 50-200 µM). However, under severe drought stress, germination remained relatively high, reaching 96% at 12% and 18% PEG (Atta et al., 2021, Atta et al., 2022), demonstrating significant tolerance compared with other *Vigna* species like mung bean, where root and shoot length frequently decrease drastically under comparable conditions (Arıkan Ceylan, 2026). Additionally, a comparative study of *Vigna unguiculata* revealed that the *VuPLD1* gene was expressed in the leaves of a susceptible cultivar under various drought conditions, whereas it remained at low levels in the drought-tolerant cultivar (Maarouf et al., 1998). Rice bean has several physiological adaptations that enable it to withstand drought. During drought, rice bean experiences oxidative stress from reactive oxygen species (ROS), which can damage cells and interfere with metabolic processes. Research on rice bean seedlings indicates that drought stress induces alterations in antioxidant enzyme activities, including catalase, ascorbate peroxidase, and guaiacol peroxidase (Atta et al., 2021). These enzymes play a crucial role in detoxifying ROS and safeguarding cellular components from oxidative damage. Furthermore, osmoprotectants such as proline accumulate under drought stress, helping maintain osmotic balance and preserving cellular hydration. Additionally, rice bean tolerates drought conditions in later growth stages through osmotic adjustment. This is achieved by accumulating *D*-ononitol, a methylated myo-inositol derivative, in leaves and stems, a process controlled by the enzyme m60MT, encoded by the *IMT1* gene (Vikram, 2022). These physiological mechanisms help rice bean sustain metabolic stability and survive in areas with inconsistent rainfall and low soil moisture. Additionally, rice bean’s deep extensive root system supports its survival and productivity in drought-prone areas by enhancing nutrient absorption and water access from deeper soil layers during dry conditions (Khatun et al., 2021; Vikram, 2022). Such adaptive features emphasise the crop’s potential role in climate-resilient farming, especially in semi-arid and marginal systems where drought often hampers productivity.

#### 4.2.2 Salinity tolerance response

A study on salinity found that rice bean varieties KRB-77, KRB-273, KRB-81, KRB-10, KRB-95, and KRB-271 demonstrate tolerance at 120 mM NaCl, which was established as the standard salt concentration screening. The tolerant varieties exhibited germination rates of 80% to 100% and high tolerance indices of 71.31 to 81.58, with susceptible indices of 0.41 to 0.60 during the seedling stage. However, some rice beans were highly affected, reducing seed germination, delaying seedling emergence, lowering growth, and decreasing plant vigour due to the high level of salinity (Nandeshwar, 2021). Also, a study on rice bean reported that physiological responses to withstand salinity during the seedling stage were observed with an increase in proline, an organic solute that protects plants from dehydration, under moderate salinity (50 mM NaCl), and the critical effects point was observed at a high level of NaCl (Atta et al., 2021). Salinity research in rice bean often shows decreases in germination rates and seedling growth as NaCl levels rise. These effects are linked to complex physiological mechanisms that govern salt stress tolerance. Initially, high salt levels cause osmotic stress, which restricts water absorption by germinating seeds and interferes with essential metabolic processes needed for seedling development. Rice bean responds to these stress effects by accumulating compatible solutes, such as proline and soluble sugars, which help regulate osmotic pressure and preserve cellular turgor. Additionally, ion-homeostasis mechanisms regulate the uptake and distribution of sodium and potassium ions, preventing harmful accumulation in sensitive cellular regions. Studies on rice bean have demonstrated that salinity stress alters biochemical markers, including increased lipid peroxidation and antioxidant enzyme activity, signalling the activation of defence systems that safeguard cells from oxidative damage during early seedling stages (Atta et al., 2021). Therefore, to identify salt-tolerant rice bean genotypes that can establish effectively in saline soils and contribute to stable crop production under climate-induced soil salinisation, it is essential to understand these physiological responses.

#### 4.2.3 Aluminium stress response

Aluminium (Al) stress significantly reduces crop yields by disrupting the absorption of water and nutrients, which are essential for vital biological processes. Rice bean tolerates Al (50 µM) stress by shedding citrate around the affected root apex and shows a pattern II response, characterised by delayed citrate efflux in the root zone, which suggests activation of genes and *de novo* protein synthesis (Yang et al., 2006). It was also confirmed that the transcription factor VuSTOP1 regulates the expression of *VuMATE2* and *VuMATE1* in response to Al stress. However, rice bean releases *VuMATE2* first in a controlled amount, followed by *VuMATE1* (Liu et al., 2018). Identifying genes responsible for Al tolerance could support the adoption of rice bean in regions susceptible to Al toxicity and facilitate the development of Al-tolerant varieties through breeding. The molecular basis of aluminium tolerance in rice bean offers a clear example of how genetic adaptations can promote climate resilience and crop enhancement. In acidic soils, aluminium toxicity poses a major challenge because soluble Al³^+^ ions inhibit root growth and nutrient uptake (Rahman et al., 2018). Rice bean has developed a detoxification mechanism that involves releasing organic acids, like citrate, from its root tips. These acids bind to toxic aluminium ions, preventing their uptake into root cells (Chauhan et al., 2021). This process involves transporter genes, such as *VuMATE1*, that are regulated by transcription factors such as *VuSTOP1*. These factors help plants initiate protective responses under aluminium stress. These genes provide important molecular targets for breeding programs for legume varieties that tolerate acidic soils (Fan et al., 2015). Since acidic soils are common in tropical and subtropical areas, integrating aluminium-tolerance traits into breeding efforts could improve the resilience and yield of rice bean in challenging environments, supporting climate-resilient agriculture. Rice bean not only exhibits tolerance to environmental stresses, including drought, salinity, and aluminium toxicity, but it also enhances resilience in nutrient-limited soils by facilitating biological nitrogen fixation (Atta et al., 2022).

#### 4.2.4 Nutrient stress adaptation and nitrogen fixation

Rice bean exhibits adaptability to nutrient-limited soils through symbiotic nitrogen fixation and tolerance to environmental challenges, including drought, salinity, and aluminium toxicity. Similar to other legumes, rice beans develop mutualistic relationships with rhizobia, a group of soil bacteria that colonise root hairs and cause the development of specialised root nodules where atmospheric nitrogen (N_2_) is transformed into ammonia (NH_3_) that is used (Simon et al., 2014). This biological nitrogen fixation reduces the need for industrial nitrogen fertilizers and offers a significant source of nitrogen in low-fertility environments. Depending on cropping patterns and soil nitrogen availability, field experiments using the ^15^N isotope dilution method have demonstrated that rice beans can derive 27–86% of their nitrogen from atmospheric nitrogen fixation (Rerkasem et al., 1988). In situations with low soil nitrogen or intercropping with cereals, where competition for soil nutrients promotes a greater reliance on biological fixation, higher amounts of nitrogen derived from fixation are typically observed (Solo et al., 2025). In addition, the diversity and efficacy of rhizobial strains associated with rice bean, particularly *Bradyrhizobium* species, which generate nitrogen-fixing nodules on rice bean roots, affect the efficiency of nitrogen fixation in rice bean (Mortuza et al., 2020). The rice bean’s symbiotic ability enables it to sustain productivity in nutrient-deficient soils and enhance soil fertility in cropping systems. As a result, rice beans’ capacity to fix atmospheric nitrogen is a crucial physiological and ecological characteristic that facilitates their cultivation in low-input agricultural systems.

#### 4.2.5 Thermal adaptability and heat stress response

The physiological processes of plants, including photosynthesis, membrane stability, and reproductive development, are adversely affected by temperature fluctuations stemming from climate change. Heat stress lowers pollen viability, hinders fertilisation, and eventually decreases seed set in legumes, which are particularly vulnerable to high temperatures during the reproductive stage. In contrast, rice bean expresses physiological traits that increase its tolerance to high temperatures. Studies have revealed the existence of photothermal-insensitive rice bean (RBL 9) genotypes that can sustain reproductive processes under thermal stress across a broad temperature range (11– 42 °C), with pollen viability rates of 72–76% (Bhanu et al., 2017). In addition, research on other *Vigna* species, such as cowpea (*V. unguiculata*) and mung bean (*V. radiata*), has shown that heat tolerance is facilitated by a variety of physiological responses, such as the regulation of photosynthetic efficiency, the activation of antioxidant defence systems that mitigate heat-induced oxidative stress, and the maintenance of membrane stability (Khatun et al., 2021). To protect cellular structures and sustain metabolic processes during periods of elevated temperature, plants employ these mechanisms. Rice bean and allied *Vigna* species may provide pollen and reproductive stability under high temperatures, making them suitable for breeding high-temperature-resistant cultivars and for cultivation in thermally stressed regions.

### 4.3 Bioactive potential of rice bean

Rice bean is rich in various phytochemicals, including phenolic acids, flavonoids, tannins, saponins, and bioactive peptides (Ahmed and Jamil, 2024). Previous research has highlighted the pharmacological and health benefits of these compounds, such as their anti-tumour, anti-atherosclerotic, and anti-inflammatory properties (Katoch et al., 2023). One study identified 35 phenolic compounds in water and aqueous methanol extracts. The major phenolic compounds identified were isoquercitrin (2.63–163.45 mg kg^-1^ DW), procyanidin B1 (3.35–106.63 mg kg^-1^ DW), catechin (0.14–72.04 mg kg^-1^ DW), rutin (0.68–58.07 mg kg^-1^ DW), taxifolin (0.17–52.85 mg kg^-1^ DW), *p*-hydroxybenzoic acid (0.53–38.85 mg kg^-1^ DW), and protocatechuic acid (0.76–23.58 mg kg^-1^ DW) (Jiang et al., 2023). Another study reported 8 phenolic compounds using 70% ethanol, with vitexin and catechin as the major phenolic compounds in the D0000090 rice bean variety, at 401.84 ± 21.57 mg kg^-1^ DW and 190.29 ± 3.69 mg kg^-1^ DW, respectively (Yao et al., 2012).

Research indicates that rice bean phenolic compounds exhibit antioxidant properties, as evidenced by several radical scavenging assays, including 3-ethyl-benzothiazoline-6-sulfonic acid (ABTS+), 2,2-diphenyl-1-picrylhydrazyl (DPPH), oxygen radical absorption capacity (ORAC), and ferric reducing antioxidant power (FRAP) (Thaipong et al., 2006). These assays demonstrated significant antioxidant activities for bound phenolic compounds extracted by base hydrolysis, with values of 90.49 and 75.74 µmol TE g^-^¹ DW for radical scavenging activity, 161.09 µmol TE g^-^¹ DW for oxygen radical absorbance capacity, and 73.04 µmol Fe(II)SE g^-1^ DW for ferric reducing ability, respectively (Jiang et al., 2023). Additionally, genotype IC-548756 showed a higher free radical scavenging activity with an average of 47.48 μMTE g^-1^ (Katoch et al., 2023). It was also reported that DPPH radical-scavenging activity ranged from 39.87 to 46.40 μmol TE g^-1^ DW, with the rice bean variety D0000874 showing the highest activity. Therefore, the total phenolic content and its bioactivities in rice beans vary greatly depending on the extraction method, environmental factors, and genotype (Yao et al., 2012; Li et al., 2019).

The phenolic and flavonoid compounds in rice bean are important not only for nutritional and health benefits but also for their functional traits that mitigate stress. These compounds are part of the plant’s antioxidant defence system, helping protect cells from oxidative damage caused by conditions such as drought, salinity, and high temperatures (Fujita and Hasanuzzaman, 2022) (Fujita and Hasanuzzaman, 2022). The phenolic and flavonoid compounds protect the plant’s cellular membranes from oxidative injury and help maintain homeostasis by scavenging reactive oxygen species (ROS) (Rao and Zheng, 2025). Research on rice bean indicates that the crop accumulates phenolics and other antioxidant metabolites under harsh environmental conditions. The accumulation of these compounds in rice bean enables the crop to survive in marginal and stress-prone environments where it is commonly cultivated. The variation in phenolic profiles amongst rice bean accessions indicates underlying genetic differences that affect secondary metabolite production (Yao et al., 2012; Jiang et al., 2023). In legumes, specifically rice bean, phenolic compounds are produced via the phenylpropanoid biosynthesis pathway, regulated by key enzymes such as phenylalanine ammonia-lyase (PAL) and chalcone synthase (CHS), as well as transcription factors that control flavonoid biosynthesis (Kumar et al., 2023). These differences in phenolic compound biosynthesis and accumulation are likely attributed to genetic diversity, resulting in diverse phytochemical composition. This means some genotypes naturally contain higher levels of beneficial compounds (Iannucci et al., 2021). Such variability offers valuable opportunities for crop improvement, including the development of rice bean varieties that are more nutritious, have high antioxidant capacity, are more resilient to biotic and abiotic stressors, and are well-suited for use as functional foods.

Conversely, legumes also contain anti-nutritional factors, such as phytic acid, saponins, and tannins, that affect the digestibility of nutrients (Samtiya et al., 2020). However, the level of phytic acid reported in rice bean, with a mean of 3.88 mg/100 g, is low compared to 519 mg/100 g in black gram (Kataria et al., 1989). Tannins, like phytic acid, hinder the absorption of minerals and nutrients by forming strong complexes with starch, cellulose, iron, and other minerals, thereby affecting digestion. It has been reported that rice beans contain 0.56% – 1.44% tannins, which aligns with Katoch’s report of 1.37% – 1.55% and with other legumes at 1.15% – 1.96% (Katoch, 2013). However, this contrasts with a study that recorded 577.95 mg/100 g of tannins in rice bean, 687.98 mg/100 g in *Vigna mungo*, 518.61 mg/100 g in *Vigna radiata*, and 394.32 mg/100 g in *Vigna unguiculata* (Katoch and Rana, 2017). The levels of tannin and phytic acid amongst rice bean genotypes suggest significant genetic variability affecting these antinutritional compounds, such that breeding approaches could leverage this diversity to optimise nutritional profiles. Additionally, processing techniques such as soaking, germination, and fermentation can reduce these substances, thereby enhancing mineral bioavailability and the overall nutritional value of rice bean products (Wu et al., 2016). In addition to their antinutritional activities, tannins from *Galliandra portoricensis* have been reported to exhibit antimicrobial activity against *E. coli*, *Staphylococcus aureus*, and *Streptococcus faecalis* (Chung et (Chung et al., 1998).

## 5 Breeding approaches for climate adaptability

### 5.1 Conventional breeding strategies

In conventional breeding, crop improvement depends on whether the crop is cross-pollinated, self-pollinated, or clonally propagated, and common methods used by breeders include mass selection, pure line selection, pedigree, bulk population, single seed descent, and backcrossing (Popelka et al., 2006). However, improvements in rice bean, based on domestication traits such as stem and leaf traits, were not achieved to a great extent through conventional breeding techniques.

A study utilised a backcross population and reported low heritability (<70%) for stem- and leaf-related traits across interspecific populations of rice bean and its wild relatives. Additionally, segregation distortion was observed in 61 markers (P<0.05, 18.7%) across the linkage groups in the intraspecific crosses. The 26 markers towards heterozygous genotypes were skewed, resulting in a higher-than-expected number of wild rice bean alleles (Isemura et al., 2010). This suggests the presence of genetic barriers affecting trait introgression. Furthermore, a significant distortion was observed in the F2 generation in a cross between rice bean and *Vigna nakashimae*. This distortion was reported across three loci, *CEDG096, CEDG105c*, and *CEDG063*, which were not mapped. The loci exhibited high distortion in the heterozygous state (P < 0.001) and were located on linkage groups 2, 6, and 11 (Somta et al., 2006). Numerous studies have shown that *Vigna* species exhibit a wide range of marker distortion, from 3.9% in *V. angularis* to 48.5% in *V. unguiculata* (Kaga et al., 2005; Isemura et al., 2010; Dachapak et al., 2018). The high level of distortion, especially in *Vigna* species, is likely to impair conventional breeding methods that rely on phenological traits, often resulting in the transfer of undesirable traits.

Conventional breeding has played a key role in *Vigna* species improvement, mainly by introducing beneficial traits such as bruchid resistance (Vishwas and Deshpande, 2018; Aidbhavi et al., 2021) and resistance to MYMV (Talakayala et al., 2022), and yield-related traits (Kaul et al., 2019). In rice bean, interspecific and intraspecific backcrossing produced hybrids exhibiting desirable domestication traits, such as longer pods, heavier seeds, extended flowering periods, taller stems, and more branches (Isemura et al., 2010). Also, the presence of phytochemicals such as phenolic acids and flavonoids indicates the potential of rice bean to adapt to a wide range of environments (Ahlawat et al., 2023). Recent research on cowpea (Simova-Stoilova et al., 2025), mungbean (Islam et al., 2024), and Bambara groundnut (Kunene et al., 2025) showed that conventional breeding has significantly improved abiotic stress tolerance in *Vigna* species by utilising genetic diversity and selecting traits such as drought resilience, enhanced root activity, and osmotic adjustment in water-scarce environments. However, the extent of breeding initiatives for rice bean remains modest compared to those for major *Vigna* crops; these accomplishments demonstrate that conventional methodologies have provided a fundamental foundation for rice bean genetic improvement, thus paving the way for a transgenic approach.

### 5.2 Genome sequencing resources in rice bean

The genomic resources available for orphan crop legumes, such as rice bean, have been significantly enhanced by recent advancements in next-generation sequencing technologies. The rice bean draft genome (~414 Mb) has been released, which will assist studies of the genes that regulate domestication traits, flowering behaviour, and seed palatability (Kaul et al., 2019). A genome-wide study identified candidate genes involved in photoperiod response, flowering regulation, and seed coat traits that are critical for adaptation across diverse agro-ecological zones (Kaul et al., 2022). Strong synteny across the genomes of rice beans, mung beans, adzuki beans, and cowpeas has been revealed by comparative genomic studies amongst *Vigna* species, suggesting that important agronomic loci are conserved throughout the genus (Kang et al., 2014; Chen et al., 2023; Dwivedi et al., 2023; Wu et al., 2024). For instance, mung bean genome sequencing revealed genes linked to stress tolerance and blooming time that may be useful for enhancing related legumes (Kang et al., 2014). Similarly, the cowpea genome project identified genetic regions associated with environmental adaptability and domestication traits (Wu et al., 2024). By identifying potential genes related to stress tolerance, nutrition metabolism, and yield attributes, these genomic resources provide useful targets for rice bean marker development and genomic breeding. Additionally, the identification of regulatory pathways linked to stress responses and secondary metabolite biosynthesis has been facilitated by integrating genomic data with transcriptomic and metabolomic analyses in legumes. This could aid in the development of rice bean varieties that are climate resilient.

### 5.3 Genome-wide association studies

The identification of marker–trait associations across diverse germplasm populations has made genome-wide association studies (GWAS) crucial instruments for dissecting complex agronomic traits. Several genomic regions associated with flowering time, and seed yield traits were identified in rice bean through GWAS conducted on 440 landrace accessions representing broad geographical diversity (Guan et al., 2022). The research shows that natural genetic variation in rice beans is shaped by their geographical distribution, suggesting that they have adapted to a variety of environmental conditions. Numerous loci linked to photoperiod sensitivity and stem determinacy were found, indicating that these characteristics are crucial for rice bean adaptability to different latitudes and climatic conditions. The GWAS results also revealed candidate genomic regions associated with seed size and yield-related traits, vital for crop enhancement. These findings align with previous genetic research in rice bean, which identified domestication loci that influence plant architecture, pod features, and seed traits (Isemura et al., 2010). Collectively, these studies suggest that many agronomic traits in rice bean may be governed by relatively simple genes mechanisms, making it easier to identify markers for breeding programs.

Comparative studies within the *Vigna* genus further affirm the importance of GWAS in legume breeding. For instance, GWAS in cowpea has pinpointed genomic regions linked to seed protein content and yield traits (Chen et al., 2023), whilst studies in mung bean have identified loci affecting flowering time and adaptation to environmental conditions (Kang et al., 2014). These genomic comparisons emphasise the potential to transfer marker information between related *Vigna* species due to their high genomic synteny. Therefore, combining GWAS with phenotypic assessments and genomic data can accelerate marker-assisted breeding and genomic selection, thereby aiding the development of high-yielding and well-adapted rice bean in various geographical locations.

### 5.4 Pangenome resources in *Vigna* species

Pangenome analysis is a crucial approach for capturing the full spectrum of genetic diversity in crop species. It involves combining genomic data from multiple accessions and distinguishes between core genes, shared by all individuals, and dispensable genes, which contribute to phenotypic variation and environmental adaptation. Unlike single-reference genomes that represent only one genotype, pangenomes provide a more comprehensive view. This approach is particularly valuable for crop improvement because many adaptive traits, such as stress tolerance and disease resistance, are linked to structural variations and presence-absence polymorphisms that may not be evident in a single reference genome (Bayer et al., 2020; Guo et al., 2024).

Extensive structural variation and gene presence-absence polymorphisms linked to domestication and adaptation have been identified in legumes by pangenome studies. For instance, pangenome investigation of soybeans revealed significant differences in gene content between farmed and wild accessions, including genes linked to environmental adaptability and agronomic features (Li et al., 2014). In a similar vein, pangenome analysis of chickpeas revealed numerous structural variations and new genes associated with domestication processes and stress responses across several germplasm groups (Varshney et al., 2021). These results show that pangenome techniques can improve the identification of genes crucial for crop development by revealing significant allelic variation not found in single reference genomes.

The increasing availability of genome assemblies and various germplasm collections provides a solid basis for future pangenome development studies within the *Vigna* genus, although full pangenome resources for rice bean are currently scarce. The development of climate-resilient rice bean cultivars and the genomic-assisted breeding of rice beans would be facilitated by identifying structural variants and novel genes associated with stress tolerance, yield stability, and nutritional traits through the construction of a rice bean pangenome. This approach would complement genome sequencing and GWAS technologies.

### 5.5 Transgenic approaches

In a rapidly growing global population expected to reach 9 billion by 2050, the production of crops with high agronomic performance and resilience to abiotic and biotic stresses is crucial for meeting nutritional demand and ensuring food security. Plant transgenic techniques, such as Agrobacterium-mediated transformation, hairy root systems, and biolistic-mediated transformation, have been employed to introduce genes that enhance crop yield and nutritional content (Biswas et al., 2022). Various gene and genome editing techniques have been also developed, and most share a common principle: using nuclease enzymes directed to specific positions in the genome to unwind the DNA double strand. The following technologies are currently in use in the field: Zinc finger nucleases (ZFNs), Meganucleases, Transcription activator-like effector nucleases (TALENs), and Clustered regularly interspaced short palindromic repeats (CRISPR/Cas9) (Liu et al., 2024). However, CRISPR/Cas9 has become more prominent than other techniques because of its simplicity in targeting multiple genes simultaneously and its lower cost.

Research on cowpea transformation using Agrobacterium-mediated methods facilitated the transformation of cowpea cotyledonary node explants. This process led to the integration of the bar gene into the plant genome, which was subsequently inherited by the progeny. Also, evaluation of genetically modified Cowpea with the *Cry1Ab* gene from *Bacillus thuringiensis* (*Bt*) revealed resistance to *Maruca vitrata*, which is a pod borer, resulting in a yield increase of 2534 kg/ha compared to the previous range of 1,414–1757 kg/ha (Addae et al., 2020; Nboyine et al., 2024). However, the transformation efficiencies in the *Vigna* species vary, such as cowpea, 1.90% (Kumar et al., 2023), green gram 4.2% (Yadav et al., 2012), and adzuki beans 12.6% (Chen et al., 2023) this indicates that variation is based on genotype and the protocol implemented (Hasan et al., 2019). The recorded low rate of transformation in legumes is linked to the intrinsic recalcitrance of many legumes to genetic transformation.

Several studies have been conducted to introduce genes into mung bean and cowpea using CRISPR/Cas9-mediated and *Agrobacterium tumefaciens*-mediated approaches, respectively, for MYMV resistance (Jha et al., 2023) and the *bar* gene, which encodes phosphinothricin acetyltransferase, responsible for detoxifying glufosinate and thereby conferring herbicide tolerance (Aasim et al., 2013). Hence, this implies that the transgenic approach has increased genetic variability in *Vigna* species more than conventional breeding (Zaidi et al., 2020). However, most research on genome editing in the *Vigna* genus has focused on cowpea and other major legumes using CRISPR/Cas9. Although there is limited research on rice bean genetic transformation, numerous rice bean-related genes, such as *VuMATE1* and *VuSTOP1*, have been utilised in other plants, such as tobacco, tomato, and *Arabidopsis*, as model systems to induce tolerance to aluminium and alter organic acid metabolism (Fan et al., 2015; Liu et al., 2018). The utilisation of heterologous transgenic methodologies verifies the functional significance of rice bean genes in conferring stress adaptation. Therefore, currently, the lack of a genetic transformation protocol for rice bean and genome-editing research creates a research gap in assessing the crop’s potential; however, its genetic similarity to Adzuki bean offers the possibility of adopting the procedure for further functional genomics and precision breeding initiatives in this underutilised crop.

Rice bean genomic resources offer an opportunity to modify genes that regulate domestication traits, including pod shattering, seed hardness, photoperiod sensitivity, and plant architecture, through genome editing. Furthermore, CRISPR-based editing of genes related to stress response pathways may improve tolerance to temperature extremes, salinity, and drought (Li et al., 2022). As a result, integrating genome-editing technologies, genome sequencing, and GWAS with pangenome resources offers a framework for identifying critical genes and accelerating the development of rice bean cultivars better suited to diverse climatic conditions.

## 6 Adoption barriers and socio-economic constraints in rice bean utilisation

Rice bean is an underutilised legume in agricultural systems, despite its adaptability to marginal environments and nutritional value. Increased adoption by farmers and consumers is constrained by a range of socioeconomic and value chain issues. Culinary traits and consumer preferences are two significant factors. Customers frequently favour more familiar legumes, such as common beans, mung beans, and lentils, in many areas where rice beans are grown because of their softer texture and shorter cooking times. Rice bean seeds are relatively hard, necessitating extended soaking and cooking times. This results in decreased consumer acceptance and household use (Bepary and Wadikar, 2018; Dhillon and Tanwar, 2018). Even though processing methods such as soaking, germination, and fermentation can enhance nutritional content and palatability, a lack of knowledge about these methods may further limit consumption.

Rice beans are subject to post-harvest losses due to pod shattering, resulting in high seed losses and contributing to their limited adoption. In mechanised and smallholder production systems, harvesting and processing are further complicated by characteristics such as indeterminate growth habit, variability in time to maturity and seed size, various seed colours, twining habit, and asynchronous flowering (Andersen, 2012). The crop’s appeal to both producers and consumers could be enhanced by addressing these constraints through breeding programs that focus on enhancing harvestability, reducing cooking time, and increasing seed quality.

Market and policy constraints further limit the development of rice bean cultivation. In comparison to other significant pulse crops, rice bean frequently lacks well-organised value chains, well-developed seed systems, and market demand, which in turn limits incentives for producers to increase production. Rice beans are mostly grown for local consumption or subsistence in many areas rather than for commercial markets. Additionally, underutilised crops often receive little funding for research, extension services, and policy assistance, which hinders the development of better cultivars and production techniques (Padulosi et al., 2002). Improving the role of rice bean in climate-resilient and sustainable agriculture could be achieved by integrating it into national crop diversification programs, promoting consumer awareness of its nutritional benefits, and strengthening value chains.

## 7 Conclusions and future directions in rice bean

**Figure 1:**
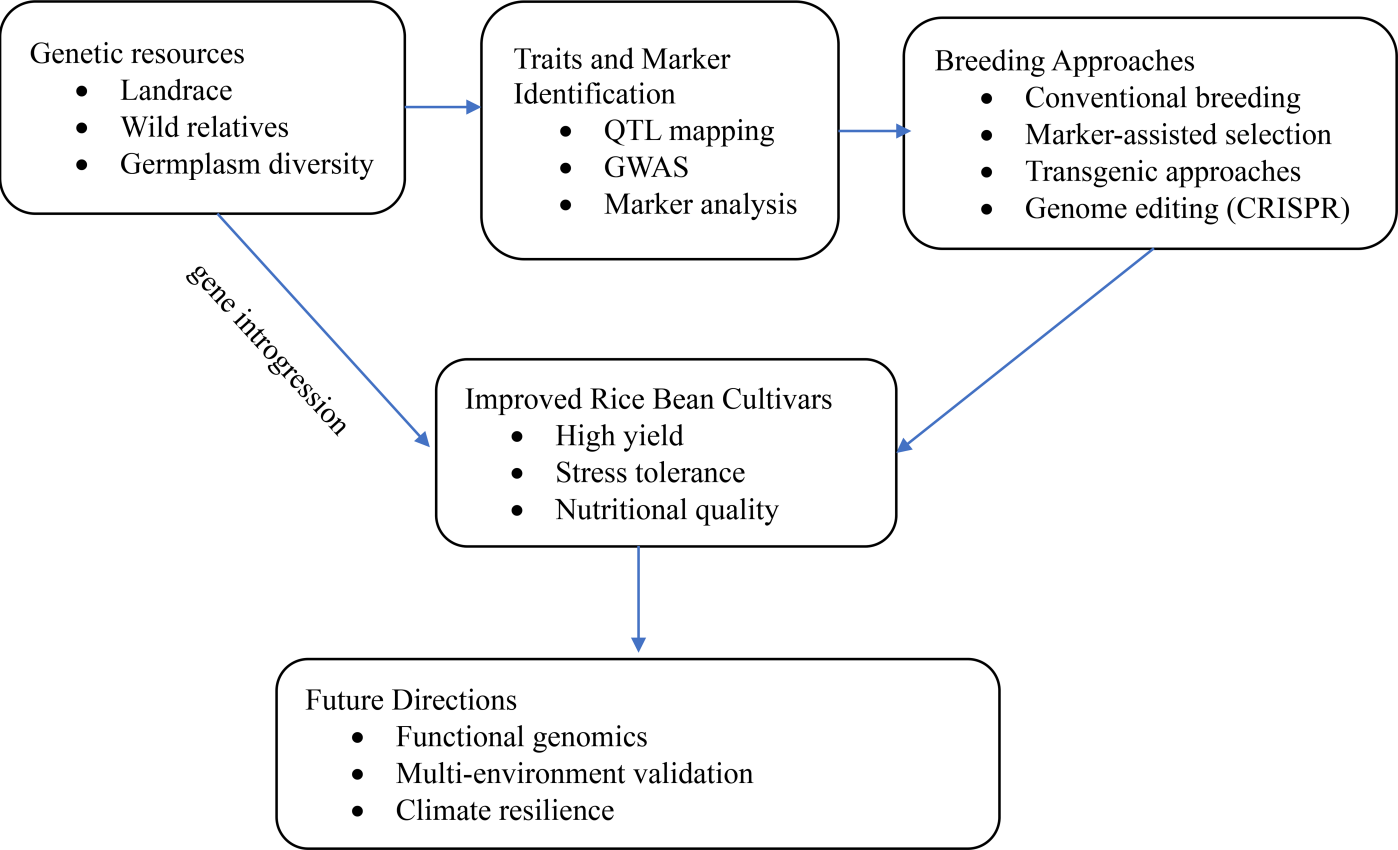
Conceptual roadmap for rice bean genetic improvement, integrating resources, trait identification, and breeding for improved cultivars.

Rice bean is considered a highly promising crop for advancing global food security and eradicating malnutrition and hunger. However, the crop remains underutilised, with barriers to adoption that hinder its widespread use. Moreover, there are few studies on the genetic transformation of legumes, especially in the *Vigna* species. Additionally, studies on the genetic transformation of rice beans using state-of-the-art approaches are not yet available. However, the limited studies on other related *Vigna* varieties can serve as a roadmap for further exploration of rice bean’s genetic and bioactive potential (**Figure 1**). This can improve its utilisation and help increase farmers’ income, especially in regions facing prolonged drought, pest, and disease outbreaks, since rice bean is known to resist various biotic and abiotic stresses. Additionally, plant breeding has evolved from traditional methods to molecular-based breeding and genetic editing techniques, enabling the identification of genes and harnessing the phytochemical compounds responsible for specific agronomic traits with greater precision. To achieve this, coordinated efforts are necessary to generate genetic resources, conserve germplasm, and conduct multi-location studies that validate improved genotypes to fulfil this potential.
